# Biogenic Nanoparticles: Synthesis, Characterization, and Biological Potential of Gold Nanoparticles Synthesized using *Lasiosiphon eriocephalus Decne* Plant Extract

**DOI:** 10.2174/2211738511666230206112537

**Published:** 2023-06-06

**Authors:** Kailas D. Datkhile, Pratik P. Durgawale, Shuvronil Chakraborty, Nilam J. Jagdale, Ashwini L. More, Satish R. Patil

**Affiliations:** 1 Department of Molecular Biology and Genetics, Krishna Institute of Medical Sciences “Deemed to be University”, Taluka-Karad, Dist-Satara, Pin, 415 539, Maharashtra, India

**Keywords:** *Lasiosiphon eriocephalus*, antioxidant activity, antibacterial activity, anticancer activity, cytotoxicity, UV-Vis spectrum

## Abstract

***Introduction*:** Recent advancements in biomedicine have revolutionized nanomedicine as a therapeutic moderator in the management of both infectious and noninfectious diseases.

***Purpose*:** In the current study we demonstrated biosynthesis of gold nanoparticles using aqueous leaf extract of *Lasiosiphon eriocephalus* as a capping and reducing agent and evaluation of their antioxidant, antibacterial, and anticancer properties.

***Methods*:** The biosynthesized LE-AuNPs were characterized by UV-Vis spectrophotometry, SEM, TEM, XRD, FTIR, DLS, and Zeta potential analysis. The antibacterial activity was checked by a minimum inhibitory concentration assay. The anticancer potential of biogenic LE-AuNPs was checked by cytotoxicity and genotoxicity assay against HeLa and HCT-15 cells.

***Results*:** The characteristic surface plasmon resonance peak of the colloidal solution at 538 nm by UV-Vis spectrum confirmed the formation of LE-AuNPs in the solution. The SEM, TEM, and XRD revealed 20-60 sized hexagonal and crystalline LE-AuNPs. The LE-AuNPs displayed significant inhibition potential against DPPH and ABTS radicals *in vitro*. The LE-AuNPs demonstrated significant antibacterial potential. The results of cytotoxicity interpreted that biogenic gold nanoparticles exhibited strong dose and time-dependent cytotoxicity effect against selected cancer cell lines where IC50 of LE-AuNPs required to inhibit the growth of HeLa cells after 24 h and 48 h exposure were 5.65± 0.69 µg/mL and 4.37±0.23 µg/mL respectively and that of HCT-15 cells was 6.46 ± 0.69 µg/mL and 5.27 ± 0.34 µg/mL, 24h and 48h post-exposure respectively.

***Conclusions*:** Findings from this study revealed that gold nanoparticles synthesized using *L. eriocephalus,* showed remarkable antioxidant, antimicrobial, and extensive cytotoxicity and genotoxicity activities.

## 1 INTRODUCTION

Recent advancements in biomedical research have revolutionized nanomedicine as a therapeutic moderator in the management of both infectious and non-infectious diseases. This emergence of novel nanobiomedicine as a promising therapeutic tool has increased its scope in the strategic development of new drugs. Metallic nanoparticles have created significant attention at the scientific level because of their versatile biological and medical applications [1, 2].

Nowadays, silver and gold nanoparticles are more commonly studied for their synthesis and wide range applications in various fields [2-5]. However, limited information is available on biosynthesized gold nanoparticles and their biological activities. Researchers across the world have screened biogenic gold nanoparticles for their variety of therapeutic applications like antioxidant [6-8], antimicrobial [9-11], and anticancer efficacy [12-14]. Thus, nanoparticles have immense biological importance in biomedicine because of their positive impact on human health. Biological synthesis is significantly attractive and widely utilized in nanotechnology for the preparation of a variety of homogenous nanoparticles because of its non-toxicity, biocompatibility, and eco-friendly nature [15, 16]. Although the biosynthesis procedure is widely utilized for the synthesis of nanoparticles, there remain challenges in plant-mediated biosynthesis approach where possible secondary metabolites and mechanism responsible for the bio-reduction process is not fully understood. Though biogenic gold nanoparticles synthesized from medicinal plants are extensively studied for their biomedical applications, their mechanism of action is still obscure and there remained insufficient evidences on any of the nanoparticles synthesized using traditional endangered medicinal plant species occurring in the hilly Western Ghats region. Some medicinal plants distributed in the hilly region of Maharashtra were screened for the biosynthesis of metal nanoparticles and their biological potentials [17-19] while others remained unresearched.

Therefore, in the current study, we intended to explore the biological potential of biogenic gold nanoparticles synthesized using *Lasiosiphon eriocephalus Decne* leaf extract. *Lasiosiphon eriocephalus* is one such rare medicinal plant with distinctive remedial properties and is scattered throughout India, mostly in southern geographic regions. Earlier studies revealed the presence of active metabolites distributed in all parts of this plant which acquired antioxidant, antimicrobial, and cytotoxicity activities [20]. In earlier studies, we demonstrated that biogenic silver nanoparticles synthesized using *L. erioocephalus* showed strong antimicrobial and cytotoxic activities. However, to the best of our knowledge, no study has reported the synthesis of gold nanoparticles using *L. eriocephalus* plant extract with their biological potentials. Therefore, in this study, we attempted biological synthesis of gold nanoparticles using *L. eriocephalus* leaf extract (LE-AuNPs) and confirm their antioxidant, antimicrobial, cytotoxicity, and genotoxicity properties. *In vitro* antioxidant potential of LE-AuNPs was demonstrated by DPPH and ABTS radical scavenging assays. The antimicrobial activity of LE-AuNPs was studied by minimum inhibitory concentration (MIC) assay against Gram-positive *Staphylococcus aureus,* and Gram-negative *Escherichia coli, Klebsiella pneumoinae, Pseudomonas auregionosa* bacteria. The cytotoxicity activity of LE-AuNPs was studied against human colon carcinoma (HCT-15) and human cervical adenocarcinoma (HeLa) cell lines by 3-(4, 5-dimethylthiazol-2yl) 2-5-diphenyltrazolium bromide (MTT) assay and the genotoxicity was addressed through DNA fragmentation assay.

## 2 MATERIALS AND METHODS

### 2.1 Preparation of Aqueous Leaf Extract of *L. eriocephalus* for the Synthesis of Nanoparticles

Aqueous extract of *L. eriocephalus* was prepared by Soxhlet extraction of 10 grams (gm) of leaf powder in 250 milliliters (mL) of double distilled water up to 15 cycles was used for biosynthesis of gold nanoparticles.

### 2.2 Biosynthesis and Purification of Gold Nanoparticles

Gold nanoparticles were synthesized by adding 100 mL of aqueous plant extract of *L. eriocephalus* to 900 mL of 1 mili molar (mM) Gold (III) chloride trihydrate (HAuCl_4._ 3H_2_O) solution. The preparation was then incubated at 80°C in dark and monitored for change in colour indicating synthesis of gold nanoparticles. After completion of biosynthesis, LE-AuNPs were centrifuged at 10,000 rpm for 10 minutes and washed several times with sterile deionized water to remove unwanted traces of contaminants followed by re-dispersion of the pellet in sterile distilled water for further characterization.

### 2.3 Characterization of Biogenic LE-AuNPs

The reduction of gold chloride using aqueous plant extract was monitored for 10, 30, 45, and 60 minutes time periods and the appearance of purple color indicated the formation of AuNPs. Primary characterization of biosynthesized LE-AuNPs was carried out using UV-Visible spectroscopy by measuring the spectrum of the reaction mixture at 200-800 nm wavelengths by sampling the aliquots withdrawn from the reaction mixture at different time intervals. LE-AuNPs were further characterized by SEM, TEM, XRD, FTIR, DLS, and Zeta potential analysis at Sophisticated Analytical Instrument Facility at Sophisticated Test and Instrumentation Centre (SAIF-STIC), Cochin University, Kerala.

### 2.4 Antioxidant properties of LE-AuNPs

Antioxidant activity of LE-AuNPs was characterized by *in vitro* DPPH & ABTS assays. DPPH assay consisted of a mixture of freshly prepared 1 mL DPPH (0.1 mM) solution in methanol with different concentrations (5, 10, 20, 40, 60, 80, and 100 μg/mL of LE-AuNPs in a final volume of 0.1 mL in distilled water. After 30 minutes of incubation of the reaction mixture at room temperature, the absorbance was measured at 517 nm by using a UV-Visible 1800 spectrophotometer (Shimadzu). ABTS radicals were produced by reacting ABTS (7 mM) in potassium persulfate (2.4 mM) and incubating the mixture at room temperature in dark for 16 hr. Different concentrations of LE-AuNPs in a final volume of 0.1 mL were added and allowed to react with 1 mL of ABTS for 30 minutes and the absorbance was recorded at 734 nm after incubation. The percentage of DPPH and ABTS radical scavenging activity was calculated using the formula: Percentage inhibition (%) = (A0-A1) / A0) × 100, where: A0 is the Absorbance of control and A1 Absorbance of the test. The results were compared with Butylated hydroxy toluene (BHT) as standard.

### 2.5 Evaluation of Antimicrobial Properties of LE-AuNPs

Antimicrobial activity of LE-AuNPs was performed against pathogenic Gram-positive bacteria *Staphylococcus aureus* (*S. aureus*) (ATCC^®^ 29213™) and Gram-negative bacteria, *Escherichia coli* (*E. coli*) (ATCC^®^ 25922™), *Klebsiella pneumoniae* (*K. pneumoniae*) (ATCC^®^ 700603™) and *Pseudomonas aeruginosa* (*P. aeruhinosa*) (ATCC^®^ 2617™) by minimum inhibitory concentration (MIC) assay.

### 2.6 *In vitro* Evaluation of Cytotoxicity Properties of LE-AuNPs

The biogenic LE-AuNPs were tested for cytotoxicity against HeLa and HCT-15 cells determined by the MTT assay. To study the inhibitory effects of LE-AuNPs, ten thousand (1 x 10^4^) cells in 200 μL of RPMI-1640 medium per well were seeded in 96 well plates and incubated at 37°C, 5% CO_2_. After 24h incubation, cells were exposed to different concentrations (2.5, 5, 7.5, 10, 12.5, 15, and 20 µg/mL) of nanoparticles in a culture medium without FBS and incubated further for 48 h at 37°C & 5% CO_2_. Thereafter, the percentage inhibition of cell proliferation of treated and untreated control cells was determined by the MTT assay by measuring absorbance at 560 nm. To check the effects of LE-AuNPs on the cell morphology of HeLa and HCT-15 cells, the cells were observed under a phase-contrast microscope.

### 2.7 Evaluation of the Genotoxic Activity of LE-AuNPs

In order to check the toxic effects of LE-AuNPs on cell DNA, a DNA fragmentation assay was performed. In brief, 1x10^6^ cells (HeLa & HCT-15) in 6 well plates were treated with (2.5, 5, 10, 15, and 20 µg/ml) concentrations of LE-AuNPs along with untreated controls and incubated at 37°C in 5% CO_2_. After 24 h of treatment, the culture medium was removed and cells were harvested by trypsinization (2.5% Trypsin, 0.02% EDTA) and washed with HBSS several times thereafter treated with 0.3 mL of cell lysis buffer containing 10 mM Tris-HCl, pH 7.5, 1 mM EDTA, 0.2% triton X- 100, 0.5% SDS. The cell lysate was incubated with 0.5 mg/mL of RNase A at 37°C for 1 h and 10 mg/mL proteinase K for 1 h at 55°C. Fragmented DNA was precipitated by adding 1/10^th^ volume of 5 M sodium chloride and an equal volume of isopropanol at room temperature. After 1h incubation, the suspension was centrifuged at 12,000 rpm for 30 min at 4°C followed by DNA pellet wash by 70% ice-cold ethanol and resuspended in an appropriate volume of Tris-EDTA buffer (T_10_E_1_; pH 8.0). For qualitative analysis, an equal amount of DNA was checked on 1.5% (*w/v*) low EEO agarose gel containing 1μg/mL ethidium bromide at 80V constant voltage, and the DNA fragments were visualized by exposing the gels to UV transilluminator followed by photography in gel documentation system (BioRad Laboratories, USA).

### 2.8 Statistical Analysis

All statistical analyses were performed with SPSS for windows version 11.0 software. The IC_50_ values with a 95% confidence interval are reported as Mean ± SEM of three independent experiments. Student's t-test were performed to examine the significant differences between means of three independent experiments from LE-AuNPs treated and control samples.

## 3 RESULTS

### 3.1 Biosynthesis and Characterization of Biogenic LE-AuNPs

**Fig. (1):**
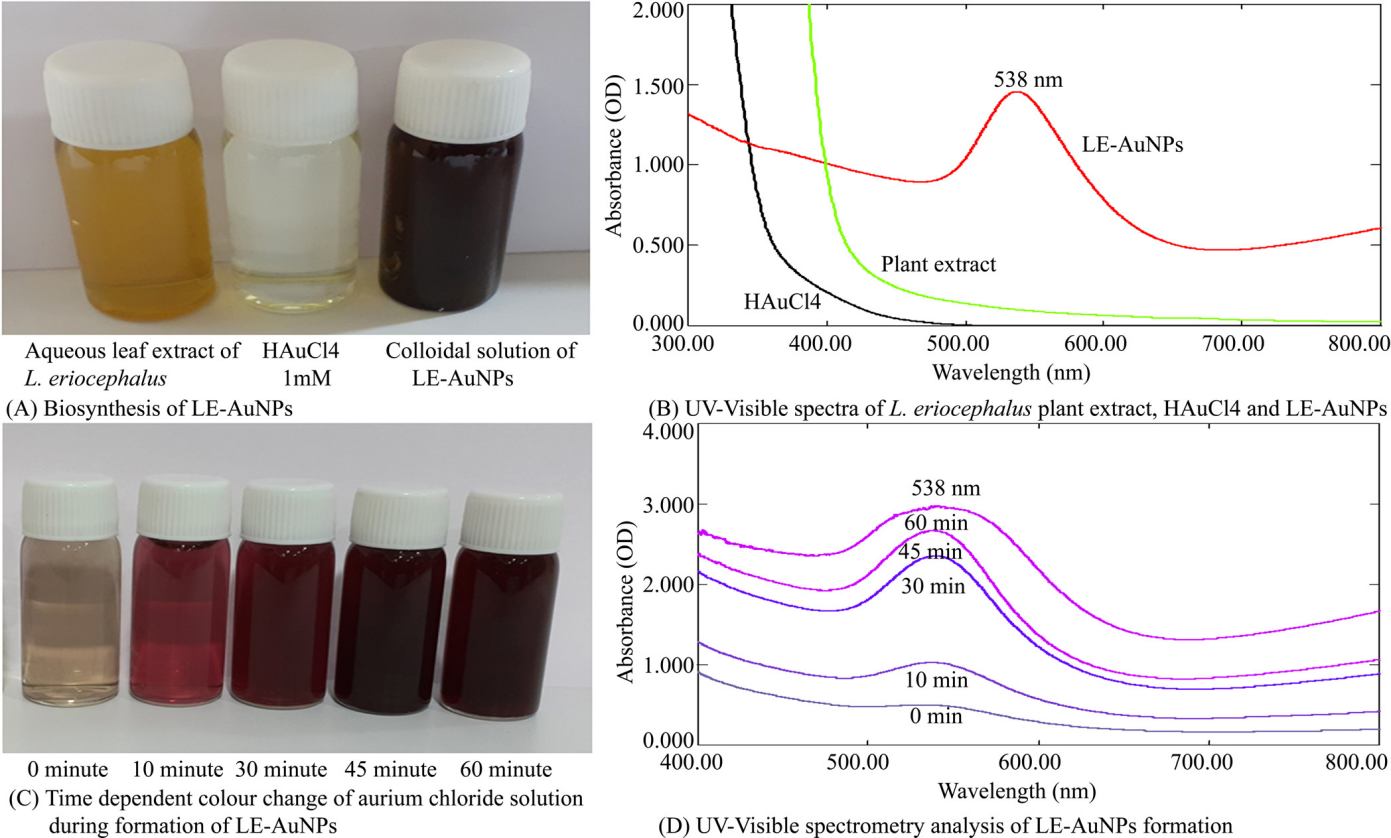
Biosynthesis of LE-AuNPs; (**A)** *L. eriocephalus* plant extract, the sample of 1 mM HAuCl4, and colloidal solution of LE-AuNPs. (**B**) UV-Visible spectra of *L. eriocephalus* plant extract, HAuCl4, and LE-AuNPs (200 nm-800 nm). (**C**) Transformation of the color of reaction mixture containing *L. eriocephalus* leaf extract and Aurium chloride solution at various time periods (0 to 60 minutes). (**D**) Time-dependent UV-Visible spectrometry analysis of the formation of LE-AuNPs at various time periods.

**Fig. (2):**
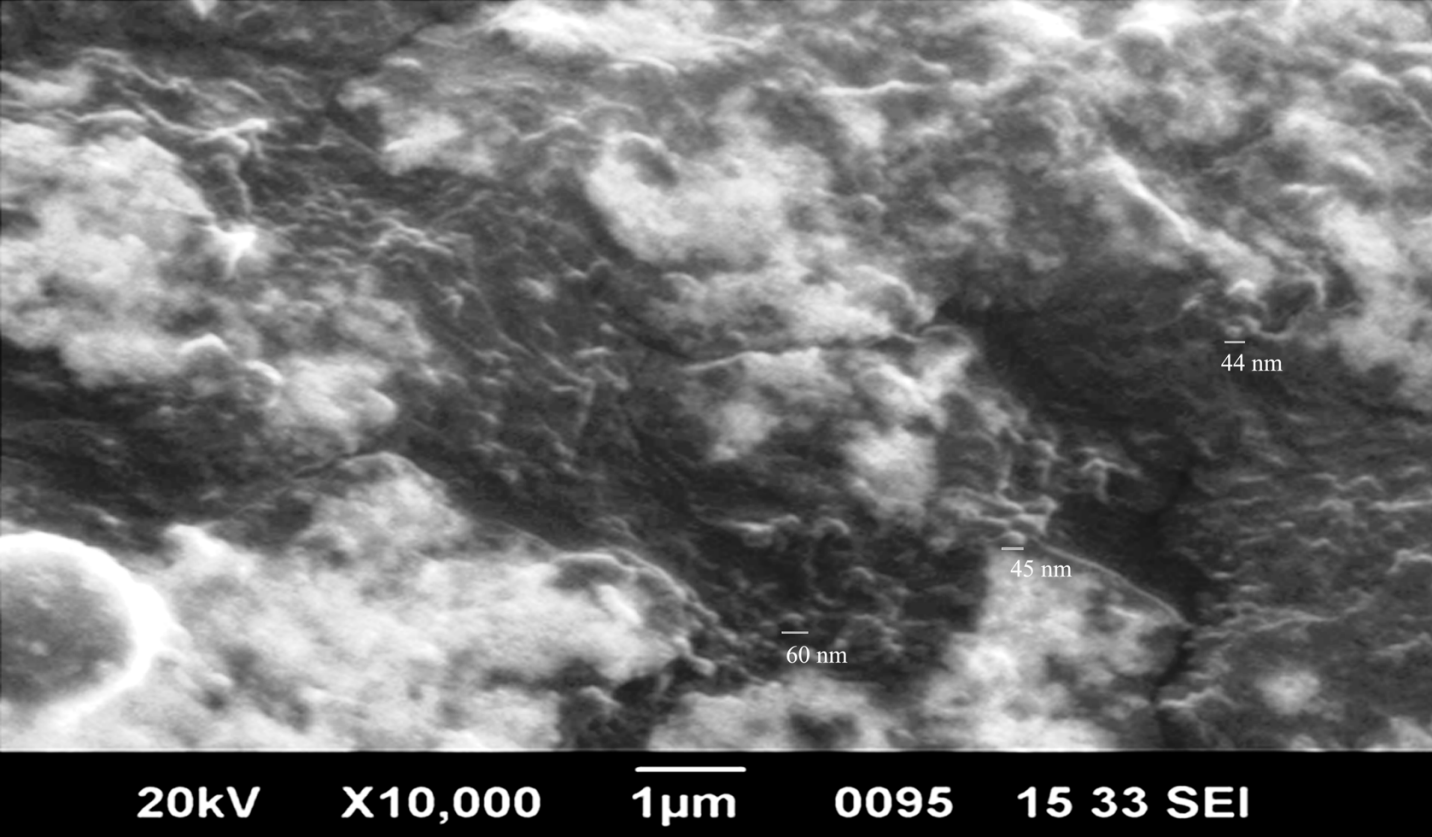
LE-AuNPs SEM microscopic view of LE-AuNPs.

**Fig. (3):**
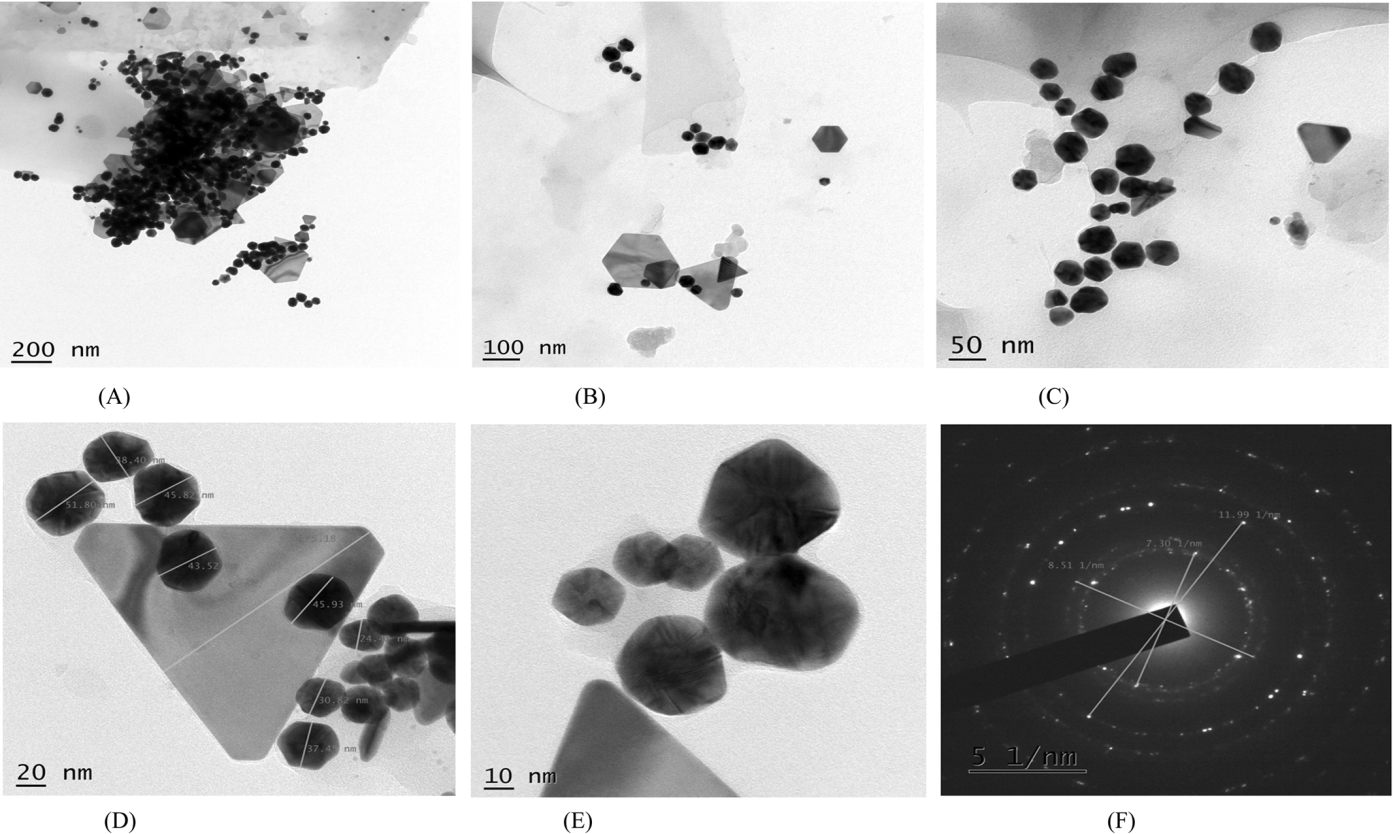
(**A-F**) LE-AuNPs TEM micrograph of LE-AuNPs and selected area electron diffraction (SAED) pattern at the right corner.

**Fig. (4):**
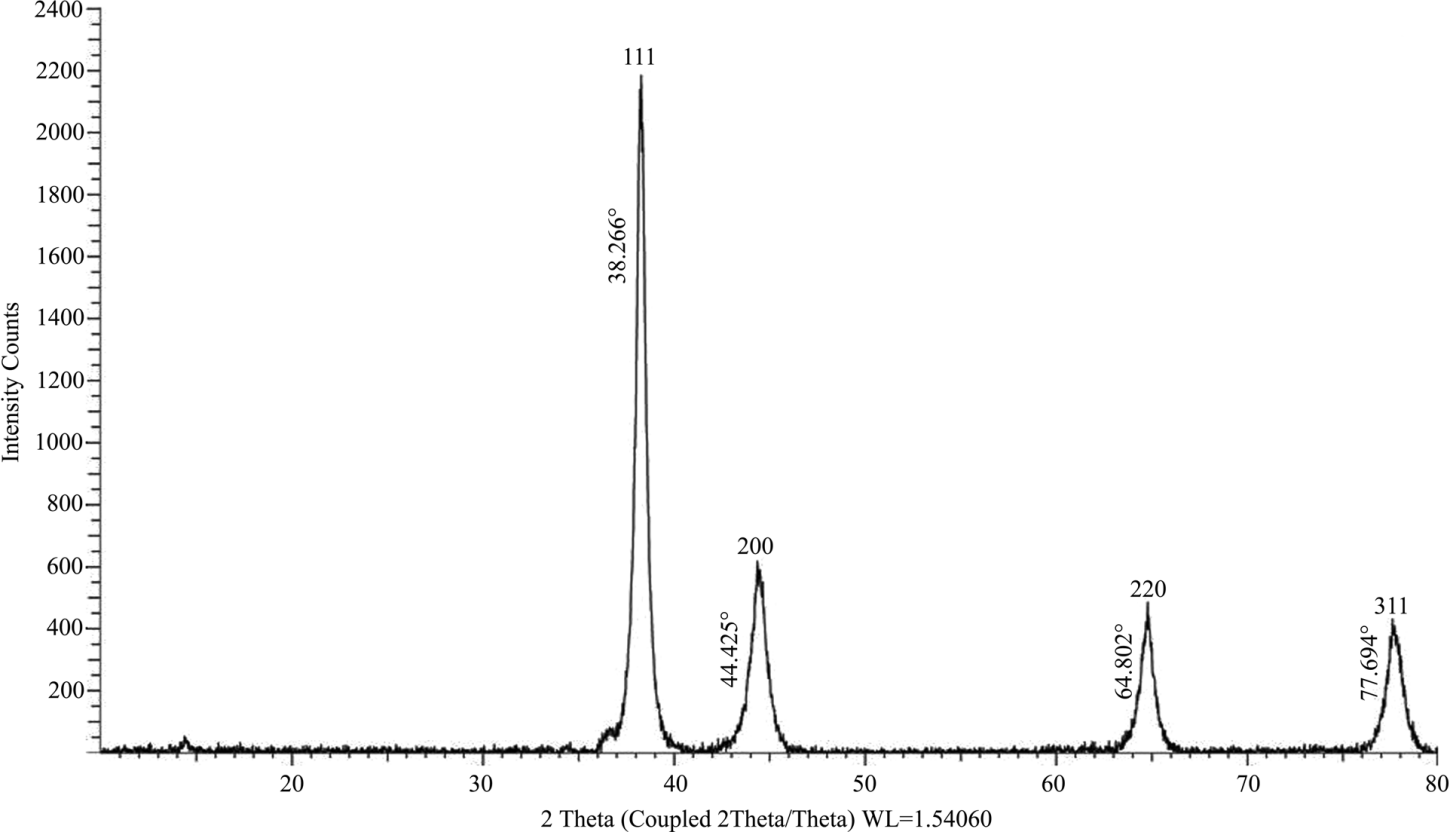
LE-AuNPs XRD pattern of LE-AuNPs synthesized using *L. eriocephalus* leaf extract.

**Fig. (6):**
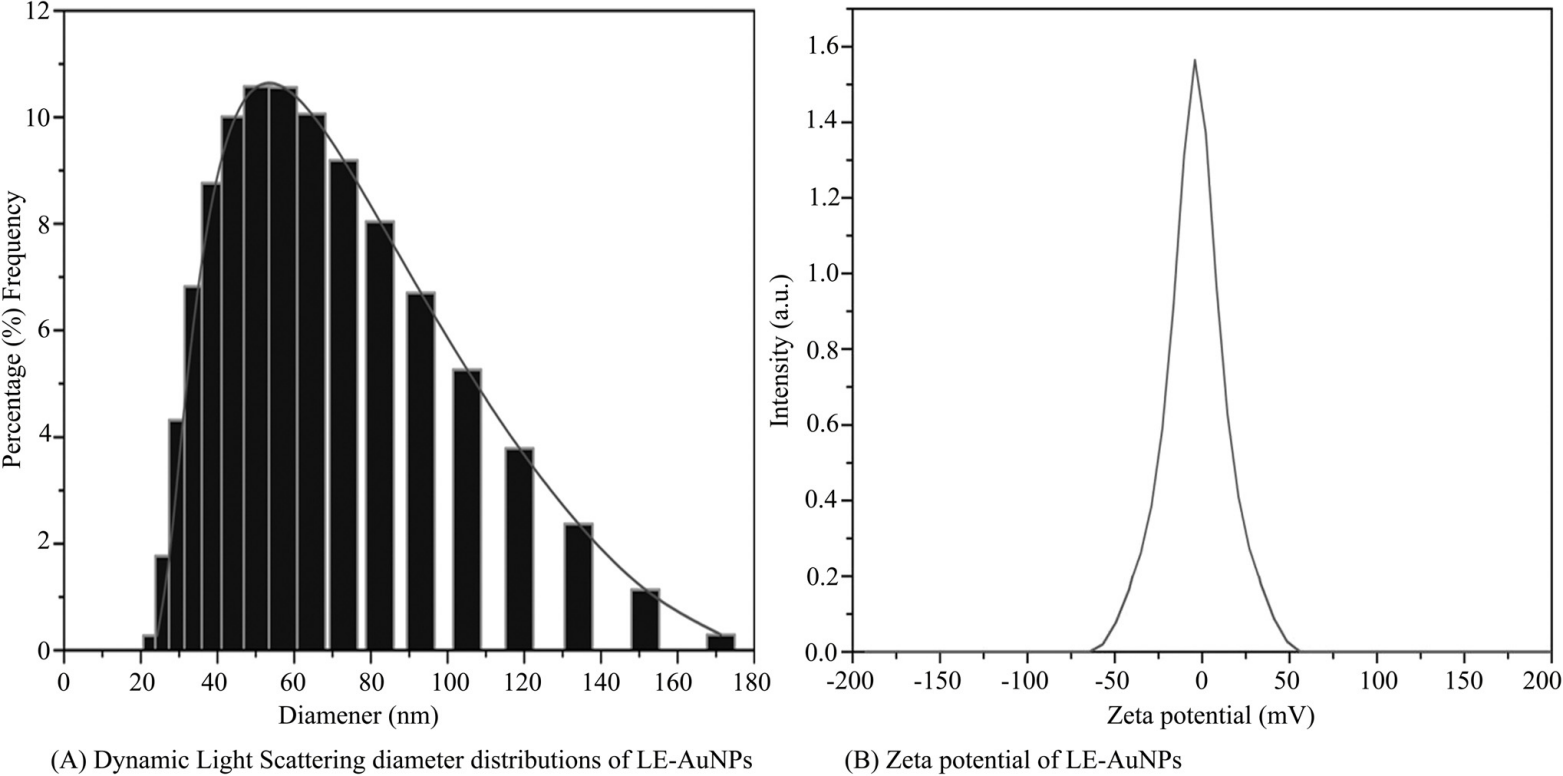
(**A**) Dynamic light scattering diameter distribution (**B**) Zeta potential of LE-AuNPs.

Biogenic synthesis of gold nanoparticles (Fig. **1**) using aqueous leaf extract of *L. eriocephalus* was demonstrated by an apparent color transition of aurum chloride solution from yellow to purple after incubation at 80°C in dark (Fig. **1A**). The initiation of color change started from pale yellow to purple after 10 min and dark purple color was observed after 60 minutes of incubation of the mixture of *L. eriocephalus* and gold chloride at 80°C under continuous shaking condition (Fig. **1C**). The change of yellow colored solution to purple colour confirmed the synthesis of gold nanoparticles which could be triggered by phytoconstituents present in the aqueous leaf extract. Further, primary confirmation of nanoparticle synthesis was done by UV-Visible spectroscopy where the colloidal solution of LE-AuNPs represented absorption maxima peak at 538 nm (Fig. **1B**. In addition, time-dependent bio-reduction of gold ions into AuNPs in colloidal solution was monitored at 15 minutes time intervals for 60 minutes (10 min, 30 min, 45 min, and 60 min) (Fig. **1D**). The formation of LE-AuNPs was started after 10 minutes and completed after 60 minutes of incubation. Scanning electron microscopy revealed information about the surface morphology of the LE-AuNPs. The SEM results established the distribution of gold nanoparticles of various sizes and shapes (40-60 nm) (Fig. **2**). The morphology, size, and shape of biosynthesized LE-AuNPs were determined by TEM which revealed a 20-50 nm size range and polygonal nanoparticles (Fig. **3A-F**). Structural analysis of biogenic LE-AuNPs was carried out through the XRD spectrum which showed characteristic peaks at 38.26°, 44.42°, 64.80° and 77.69° in the 2θ values corresponded to the lattice planes 111, 200, 220 and 311 of gold confirms the crystalline nature of biogenic LE-AuNPs (Fig. **4**). The highest diffraction peak of LE-AuNPs at 2θ angle *i.e*, 38.26° indicated the growth direction of crystals towards lattice plane 111. The FTIR pattern of the *L. eriocephalus* leaf extract and biogenic LE-AuNPs was recorded in the range of 500-4000 cm-1 to reveal the interaction of nanoparticles with active biomolecules involved in capping and stabilization. The LE-AuNPs exhibited FTIR peaks at 3439, 2923, 2853, 1631, 1382, and 1047 cm^-1^ and LE plant extract showed FTIR peaks at 3449, 2088, 1637, 601 cm-1 which are represented in Figs. (**5A** and **B**). A first peak at 3439 (LE-AuNPs) and 3449 cm^-1^ (LE-PE) denoted the stretching vibration of the O-H group of alcohol and phenol. The second peak shift 2923 - 2853 cm^-1^ represents the C-H stretching vibration of an alkyl group. A third peak located at 1631 cm-1 (LE-AuNPs) and 1637 cm^-1^ (LE-PE) confirmed the stretching mode of the carboxyl group. A peak located at 1047 cm-1in biogenic AuNPs arises due to the C-OH vibration. In addition to the TEM imaging, the difference in the size of LE-AuNPs and the thickness of the capping or stabilizing agent surrounding the metallic nanoparticles was also determined and confirmed by dynamic light scattering (DLS) (Fig. **6A**). The particle size distribution showed the range of particle size of LE-AuNPs between 40 to 60 nm. Similarly, the surface charge and stability of the colloidal solution of AuNPs were also checked with their zeta potential value where the biosynthesized AuNPs showed a zeta potential of -3.2 mV which indicated synthesis of negatively charged nanoparticles (Fig. **6B**). The lower zeta potential value of LE-AuNPs could be because of polar functional groups of organic molecules present in the *L. eriocephalus* leaf extract. The stability of biosynthesized LE-AuNPs was observed for more than three months with no change in color or aggregation which could be because of the steric effect produced by phytoconstituents adsorbed on the surface of nanoparticles.

### 3.2 Antioxidant Potential of Biogenic LE-AuNPs

**Fig. (7):**
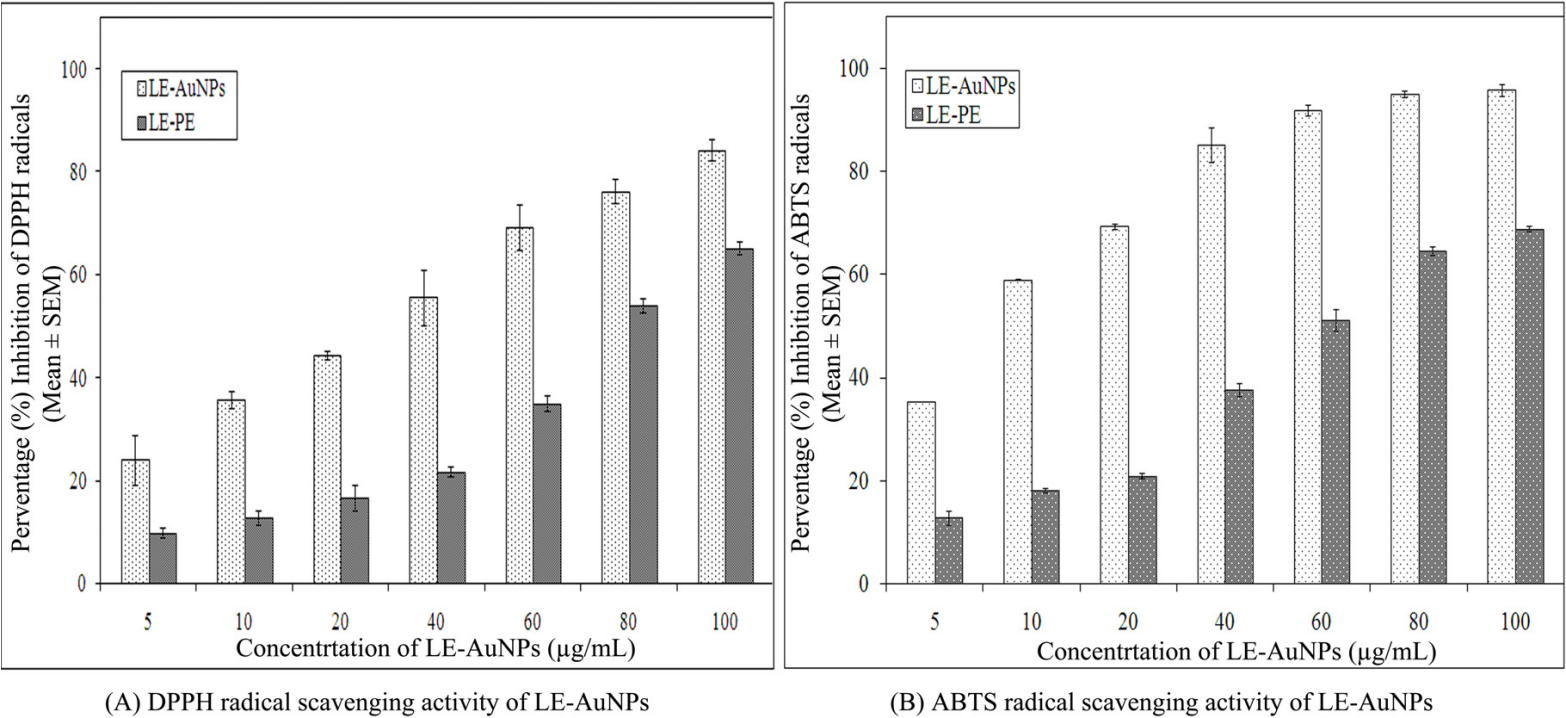
Representative histogram showing (**A**) DPPH and (**B**) ABTS radical scavenging activity of different concentrations of LE-AuNPs. The data represent the percentage inhibition of radicals *in vitro*. The results represent the means of three independent experiments, and the error bars represent the standard error of the means.

The results of the antioxidant potential of biosynthesized LE-AuNPs were confirmed by *in vitro* DPPH and ABTS radical scavenging assays. We observed significant DPPH radical scavenging activity of LE-AuNPs in a concentration-dependent manner as shown in Fig. (**7A**). LE-AuNPs exhibited higher antioxidant potential with a range of 35.84% -84.31% inhibition of DPPH radical towards 10-100 µg/mL concentrations of AuNPs. When we compared IC_50_ values of LE-AuNPs with leaf extract of *L. eriocephalus*, interesting results were noted, where IC_50_ of LE-AuNPs was reported to be 24.15 µg/mL which was significantly lower than that of aqueous leaf extract (184.07 µg/mL). Further, the ABTS radical scavenging activity of LE-AuNPs was addressed *in vitro* and the results indicated comparatively higher ABTS radical scavenging potential of LE-AuNPs with the range of 58.96% to 95.80% inhibition of ABTS radicals when exposed to 10-100 µg/mL concentrations of AuNPs (Fig. **7B**). IC_50_ values of LE-AuNPs for the inhibition of ABTS radicals were noted to be 8.88 µg/mL which was comparatively much lower than that of aqueous leaf extract (62.24 µg/mL). Thus, the results highlighted the significant radical scavenging potential of biologically synthesized gold nanoparticles as compared to that of phytochemicals derived from aqueous leaf extract *L. eriocephalus*.

### 3.3 Antimicrobial Activity of Biosynthesized LE-AuNPs

The antimicrobial activity of LE-AuNPs was addressed *in vitro* by minimum inhibition concentration method against Gram-negative bacteria species *E. coli, P. aeruginosa, K. pneumoniae,* and Gram-positive *S. aureus.* Different concentrations of LE-AuNPs (0.01, 0.02, 0.04, 0.06, 0.08, and 0.1 mg/mL) were tested on Muller Hinton media. The results of the antibacterial activity of biogenic LE-AuNPs were represented in Fig. (**8A-D**). The MIC results indicated that higher concentrations of LE-AuNPs (0.1 mg/mL) showed greater sensitivity to *E. coli*. Comparatively, *P. aeruginosa* and *K. pneumonia*e, and *S. aureus* required higher concentrations of LE-AuNPs to inhibit bacterial cell growth. When we tested the antibacterial effect of LE-AuNPs, we observed the highest percentage of bacterial growth inhibition in *E.coli* (85.67 ± 0.99) followed by *S. aureus* (65.04 ± 2.37), K. *pneumoniae* (64.64 ± 2.50) and *P. aeruginosa* strains (42.25 ± 4.95) when exposed to 0.1 mg/mL concentration of LE-AuNPs. The antibacterial potential of biologically synthesized gold nanoparticles could be due to the synergistic effect of phytoconstituents in the plant extract which may act as capping agents.

### 3.4 Anticancer Potential of LE-AuNPs

**Fig. (9):**
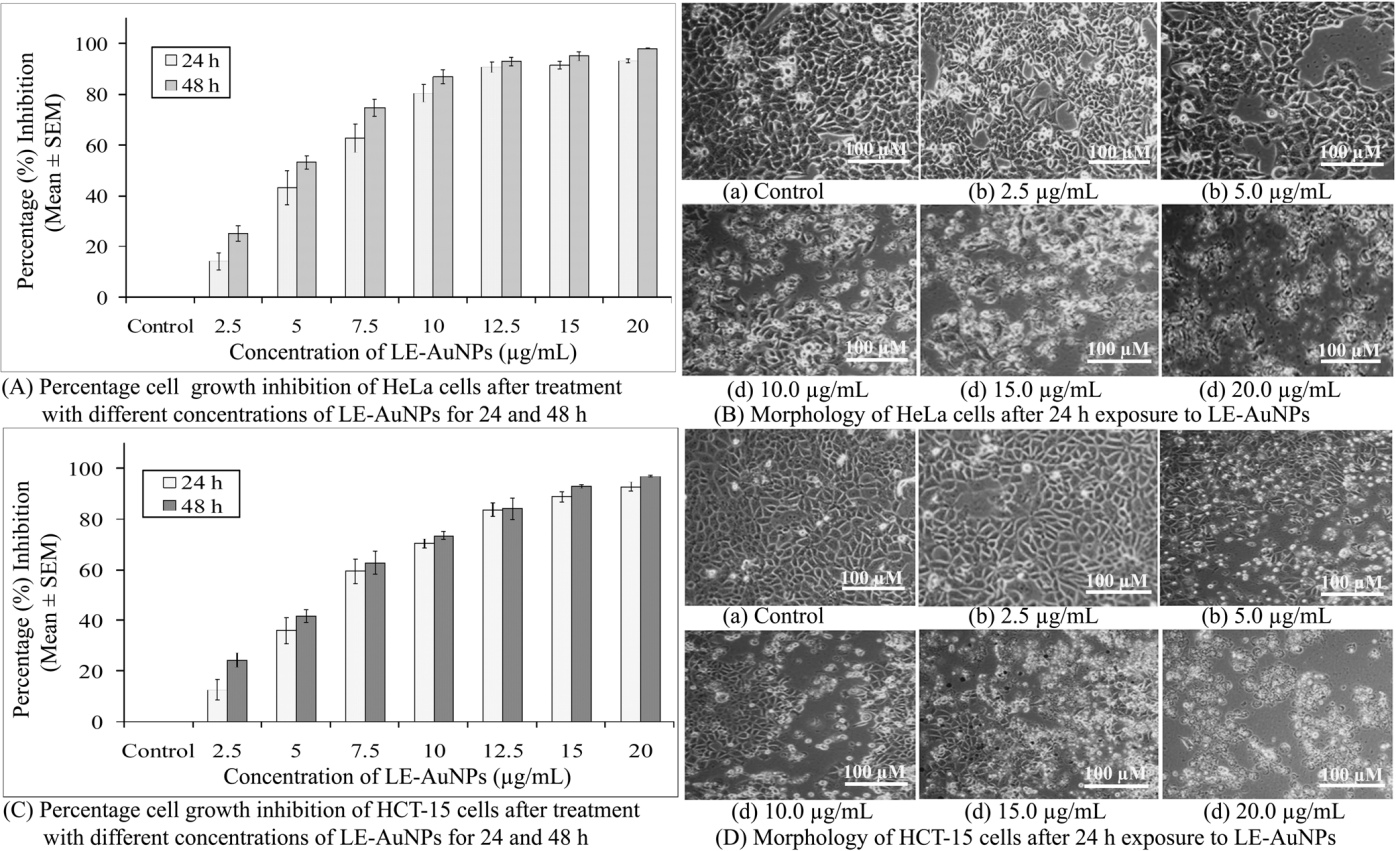
Representative histogram showing *in vitro* cytotoxicity of biogenic LE-AuNPs on (**A**) HeLa cells and (**C**) HCT-15 cells after 24 h and 48 h exposure to (2.5, 5, 7.5, 10, 12.5, 15 and 20 µg/mL) concentrations of biogenic LE-AuNPs. The results represent the means of three independent experiments, and the error bars represent the standard error of the mean. Cell morphology of (**B**) HeLa cells (**D**) HCT-15 cells after 24 h exposure to different concentrations (2.5, 5, 10, 15, and 20 µg/mL) of biogenic LE-AuNPs for 24 h. All images are taken at 20X magnification with a Carl Zeiss phase contrast microscope. Scale bars, 100 µm.

The cytotoxicity activity of biogenically synthesized LE-AuNPs was tested against cancer cell lines. The LE-AuNPs exerted significant toxicity effects on tested cancer cells in a dose-dependent manner (2.50 - 20.0 µg/mL) where cell viability decreased with an increased concentration of LE-AuNPs after 24h intervals up to 48h of exposure (Fig. **9A-D**). HCT-15 cells showed comparatively higher resistance to the AuNPs than HeLa cells. The results showed that 20 µg/mL of LE-AuNPs showed maximum cell growth inhibition (93.38 ± 0.72%) of HeLa cells after 24 h exposure and (98.17 ± 0.30%) 48h post-exposure (Fig. **9A**). The fifty percent inhibitory concentration (IC_50_) of LE-AuNPs required to inhibit the growth of HeLa cells after 24 h and 48 h exposure was 5.65 ± 0.69 µg/mL and 4.37 ± 0.23 µg/mL respectively. The minimum concentration of LE-AuNPs (2.50 µg/ml) inhibited 12.64 ± 4.05% cell growth of HCT-15 cells whereas the higher tested concentration (20 µg/ml) killed 92.86 ±1.78% of HCT-15 cells after 24 h and 96.94 ± 0.32% 48 h post-exposure (Fig. **9C**). The IC_50_ of LE-AuNPs required to kill HCT-15 cells after 24h and 48h of exposure were 6.46 ± 0.69 µg/mL and 5.27 ± 0.34 µg/mL respectively. The morphology of HeLa and HCT-15 cells was altered and exhibited a distorted appearance because of loss of membrane integrity and cytoplasmic condensation (Figs. **9B** and **D**) when treated with 10-20 µg/mL of LE-AuNPs.

### 3.5 Effect of LE-AuNPs on Apoptotic DNA Fragmentation

**Fig. (10):**
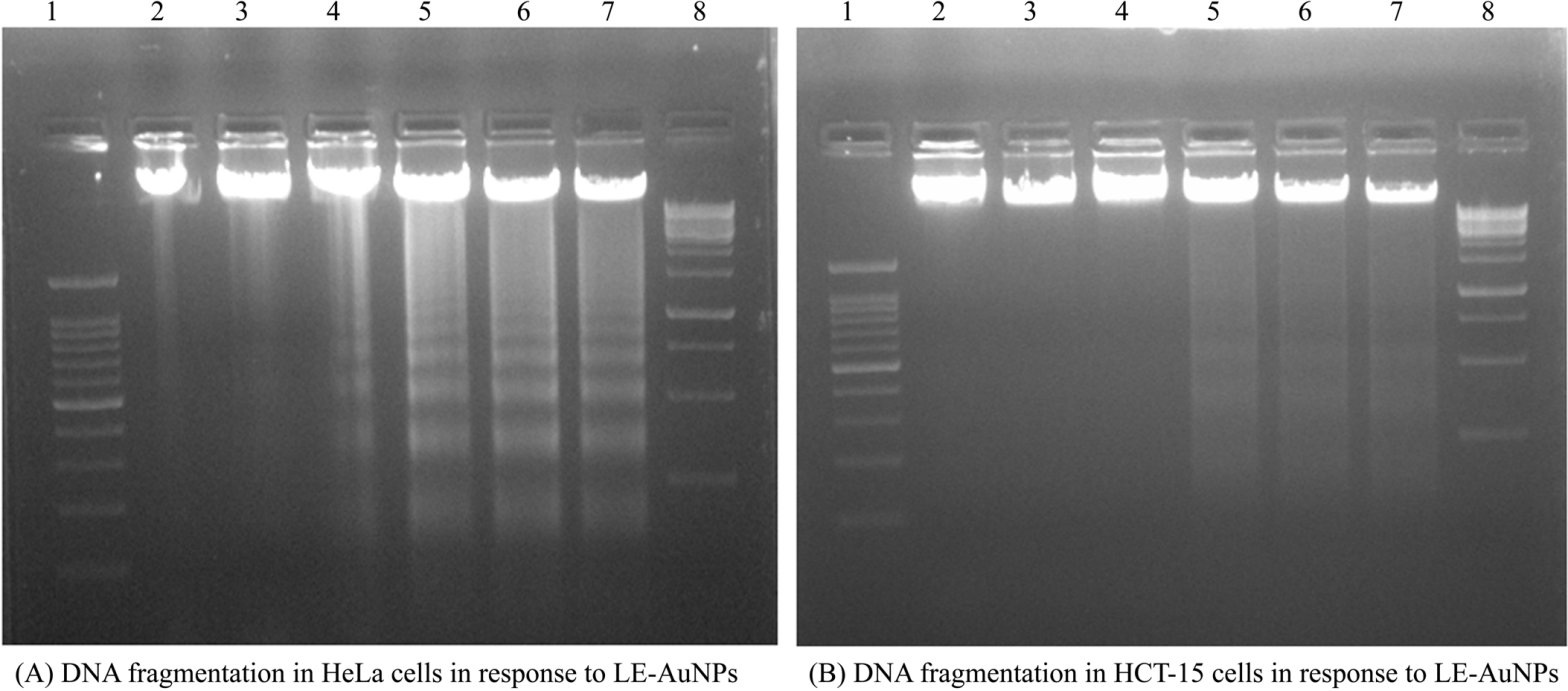
Genotoxicity of LE-AuNPs against HeLa, and HCT-15 cells. Representative agarose gel images showing DNA fragmentation in (**A**) HeLa and (**B**) HCT-15 cells treated with different concentrations of LE-AuNPs. In each representative gel, lane 1 is 100 bp DNA marker: Lane 2 is DNA from control cells followed by lanes 3, 4, 5, 6 and 7 with DNA from HeLa and HCT-15 cells treated with 2.5, 5, 10, 15, and 20 µg/mL concentrations of LE-AuNPs.

A hallmark of apoptotic cell death *i.e*., DNA fragmentation was also studied by DNA laddering assay where both HeLa and HCT-15 cells were treated with increasing concentrations (2.5, 5.0, 10, 15, and 20 µg/ml) of LE-AuNPs for 24 hours. Significant DNA fragmentation was seen in HeLa cells when exposed to 10 µg/ml and higher concentrations of LE-AuNPs (Fig. **10A**, Lane 5, 6 and 7) whereas a comparatively lower effect was noticed in HCT-15 cells with the same concentrations of LE-AuNPs where the major amount of intact DNA was seen (Fig. **10B**, Lane 5, 6 and 7). Thus, the results of agarose gel electrophoresis revealed that the HeLa and HCT-15 cells treated with different concentrations of LE-AuNPs exhibited characteristic DNA damage.

## 4 DISCUSSION

Nanomaterials are comprehensively studied for biological synthesis; nevertheless, the biosynthesis process remained vague with an opportunity to explore the mechanism of biogenic synthesis of nanoparticles and their relevance in the health management sector. In recent years, nanoparticles have captured attention as an experimental tool in diagnostic and therapeutic processes. Earlier research provided evidence that metal nanoparticles play a key role in nanotechnology because of their advanced applications in biomedicine, agriculture, industrial and environmental remediation. Certainly, it is crucial to understand the biological response of nanoparticles, especially in view of their potential biological applications. In the present study, biosynthesis of gold nanoparticles using leaf extract of *L. eriocephalus* was illustrated, where maximum absorbance (λmax) peak at 538 nm for AuNPs by UV-visible spectroscopy represents the surface plasmon resonance which was in accordance with an earlier report of biosynthesized gold nanoparticles using *Notaphodytes foetida* [19] and *Argemone mexicana* [21] leaf extracts. As observed in SEM and TEM pictures verified the hexagonal and pentagonal shape of LE-AuNPs with 20-50 nm size. Further, the crystalline structure of LE-AuNPs confirmed by XRD pattern showed characteristic diffraction peaks 38.26°, 44.42°, 64.80° and 77.69° which are typical for the gold nanoparticles formed by the reduction of Au (III) by bioorganic materials of *L. eriocephalus*. Thus the recorded FTIR spectra of both *L. eriocephalus* extract and LE-AuNPs confirmed the chemical functional groups (–OH, C–H, –C=C–) which could be the most prominent chemical bonds in the synthesis of AuNPs in the active phytoconstituents of *L. eriocephalus* acted as reducing and stabilizing agents in the synthesis of LE-AuNPs. The antioxidant defense system is active in eukaryotic cells to combat reactive oxygen species (ROS) generated by oxidative stress. The mechanism of antioxidant potential of nanomaterials can be directly studied *via* reacting with free radicals thereby reducing oxidative damage (*in vitro*) or indirectly by determining the inhibition or enhanced expression of intracellular antioxidant enzyme system [22]. DPPH and ABTS radical scavenging assays are performed *in vitro* for the determination of antioxidant potential whereas a battery of enzymes like superoxide dismutase and catalase are widely studied for *in vivo* assessment of intracellular antioxidant mechanisms. We studied the antioxidant potential of biogenic AuNPs by *in vitro* method where the results noted that biogenic LE-AuNPs showed dose-dependent radical scavenging activity against DPPH and ABTS radicals. The LE-AuNPs exhibited strong radical scavenging potential as compared to the leaf extract of *L. eriocephalus*. The significant boost in antioxidant activity of LE-AuNPs could be due to the functional groups on their surface which originated from the numerous phytoconstituents of the plant extracts used for biosynthesis in accordance with observations reported earlier by AuNPs synthesized using *Gymnema sylvestre*, *Marsellia codrifolia, Curcuma kwangsiensis* and *Persicaria salicifolia* plant extracts [23-26].

The antibacterial activity of any agent is attributed to the mechanism which includes either killing bacteria using smart or targeted antibacterial material which interferes with the function of vital bacterial components [27-29] or overcoming the conventional antibacterial resistance mechanisms. The alternate strategy employed towards combating pathogenic infections is through regulation of the micro-flora balance without antibacterial materials [30-33]. In the present study, we revealed the antibacterial properties of LE-AuNPs which showed the most significant anti-bacterial effect against infectious pathogens. When we checked the antimicrobial potential of biosynthesized AuNPs, we observed that LE-AuNPs exhibited promising antibacterial efficacies against Gram-positive and Gram-negative bacteria where the minimum inhibitory concentration was reported to be 100 µg/mL. Dose-dependent effects of biogenic gold nanoparticles synthesized using different plants are reported earlier where AuNPs synthesized using *Euphorbia hirta* contributed highly effective antimicrobial activity against *E. coli, P. aeruginosa, and K. pneumoniae* which are inhibited completely by 200 µg/mL concentration [34]. Similarly, biogenic AuNPs synthesized using *Abelmoschus esculantus* plant extract produced antimicrobial potential against *Bacillus subtilis, B. cereus, E. coli, and P. aeruginosa* [35]. The AuNPs synthesized using *Mangifera indica* seed extract showed maximum activity against bacterial pathogens including *S. aureus, E. coli, P aeruginosa,* and *K. pneumoniae* [36]. Similar results of biogenic AuNPs synthesized using diverse plant extracts were also reported against different microbes [9, 37, 38]. Cytotoxicity results of LE-AuNPs exerted a significant cytotoxic effect on cancer cells in a dose-dependent manner where inhibition of both HeLa and HCT-15 cells showed ≥ 95% cell death in HeLa and HCT-15 cells in response to biogenic LE-AuNPs. Similar cytotoxic effects of gold nanoparticles were reported *in vitro* for HeLa and HCT-15 cells in a concentration and time-dependent manner [39, 40]. This cytotoxic effect of nanoparticles is because of their easy permeability into the cells and their affinity towards different macromolecules. The cytotoxicity is evaluated by *in vitro* and *in vivo* methods to understand the impact of nanoparticles on cellular architecture and genetic material inside the cells. Previous studies on the efficacy of gold nanoparticles demonstrated that gold nanoparticles can induce cytotoxicity in cancer cells through several pathways including: Cell membrane damage, inhibition of cell proliferation, stimulation of pro-apoptotic proteins, and generation of reactive oxygen species (ROS) and reactive nitrogen species (RNS) inside the cells which can damage the cellular components and led to apoptotic or necrotic cell death [41, 42]. In our earlier studies, leaf and flower extract of *L. eriocephalus* caused cytotoxic and genotoxic effects against HeLa cells [20], likewise, the inhibition of cell proliferation was addressed in the studied cell lines in response to biogenic LE-AuNPs. Many other researchers observed the cytotoxicity of gold nanoparticles on different cancer cells [24, 43, 44]. In accordance with our earlier studies on anti-proliferative effects of biosynthesized gold nanoparticles using *Nothaphodytes foetida* and *Argemone mexicana* [19, 21], comparable toxicity results noted strong inhibitory effect on cell growth of HeLa and HCT-15 cells in response to biogenic AuNPs synthesized using *Lasiosiphon eriocephalus*. Apoptotic cell death is one of the most important mechanisms of cytotoxicity which is characterized by morphological changes like cell shrinkage, extensive blebbing of the plasma membrane, and nuclear fragmentation. Strengthening the fact, in this study we observed shrunken cell morphology and apparent DNA fragmentation in both HeLa and HCT-15 cells even when exposed to lower concentrations (10 µg/mL) of LE-AuNPs after 24 h, suggesting that the cell death is due to apoptosis. The apoptosis induced by LE-AuNPs was specifically associated with the characteristic DNA fragmentation indicating genotoxicity exerted by biogenic gold nanoparticles. Thus, to the best of our knowledge, the antimicrobial and cytotoxicity potential of AuNPs of *L. eriocephalus* has been investigated for the first time in the current study and showed anti-microbial activity against Gram-positive and Gram-negative bacteria and anticancer potential against cancer HeLa and HCT-15 cell lines in time and dose-dependent manner.

## CONCLUSION

The present study revealed the antioxidant, antibacterial, and anticancer potential of biogenically synthesized gold nanoparticles from *L. eriocephalus*. Initially, the formation of gold nanoparticles was confirmed through UV-Vis spectrum analysis, SEM, TEM, XRD, FTIR analysis, and DLS and zeta potential studies. The green biosynthesized AuNPs have shown significant antioxidant and antibacterial activities *in vitro*. The biogenic LE-AuNPs exhibited considerable cytotoxicity and genotoxicity effects against HeLa and HCT-15 cells. However, the exact mechanism of action is not very well understood, it is suggested that nanoparticles induce morphological changes in cell membranes thereby disrupting the membrane integrity and permeability ultimately leading to cell death.

## Figures and Tables

**Fig. (5):**
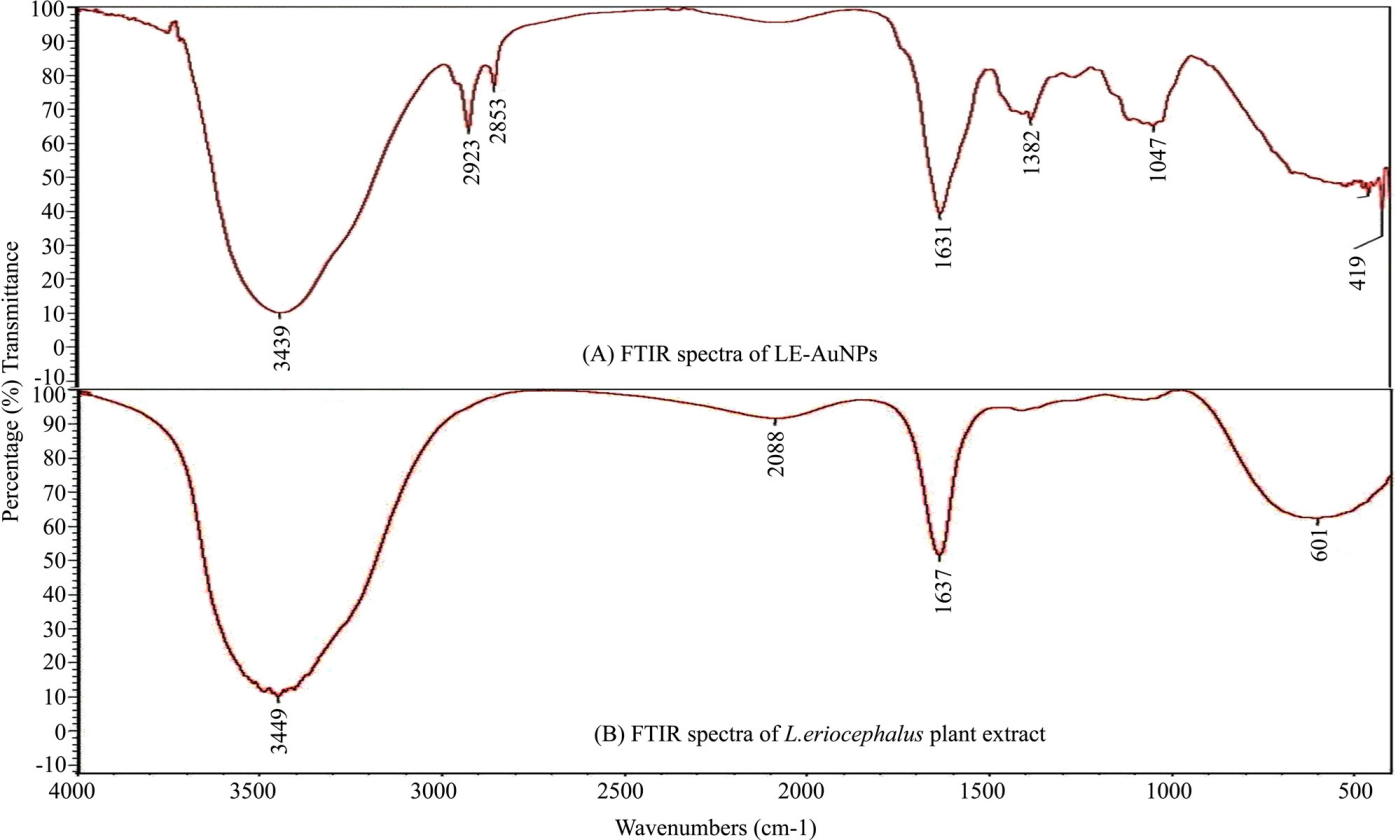
FTIR spectrum of (**A**) biogenic LE-AuNPs and (**B**) Aqueous *L.eriocephalus* leaf extract.

**Fig. (8):**
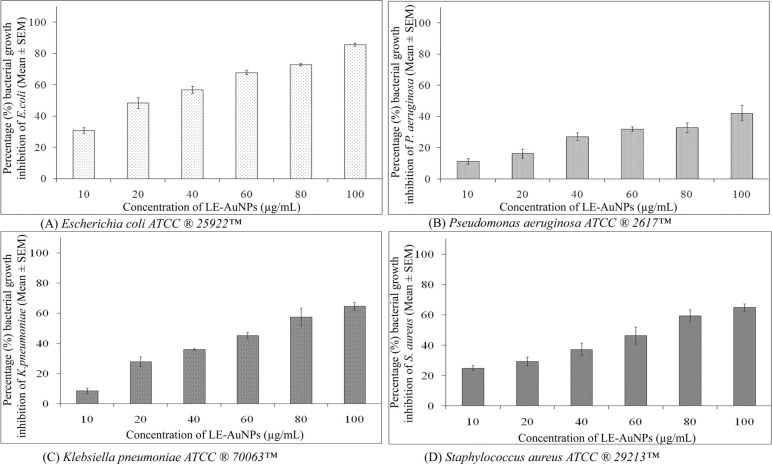
Representative histogram showing bacterial growth inhibition of (**A**) *Escherichia coli ATCC* ***^®^*** *25922™* (**B**) *Pseudomonas aeruginosa ATCC* ***^®^*** *2617™* (**C**) *Klebsiella pneumoniae ATCC* ***^®^*** *70063™ and* (**D**) *Staphylococcus aureus ATCC* ***^®^*** *29213™* exposed to different concentrations (10, 20, 40, 60, 80 and 100 µg/mL) of LE-AuNPs for 24 h. The results represent the means of three independent experiments, and the error bars represent the standard error of the mean.

## Data Availability

The data supporting the findings of the article are available within the article.

## LIST OF ABBREVIATIONS

DLS: Dynamic Light Scattering
ROS: Reactive Oxygen Species
RNS: Reactive Nitrogen Species
MIC: Minimum Inhibitory Concentration
BHT: Butylated Hydroxy Toluene

## References

[1] Rathore B., Sunwoo K., Jangili P. (2019). Nanomaterial designing strategies related to cell lysosome and their biomedical applications: A review.. Biomaterials.

[2] Yaqoob S.B., Adnan R., Rameez Khan R.M., Rashid M. (2020). Gold, silver, and palladium nanoparticles: A chemical tool for biomedical applications.. Front Chem..

[3] Lee S., Jun B.H. (2019). Silver nanoparticles: synthesis and application for nanomedicine.. Int. J. Mol. Sci..

[4] Lee K.X., Shameli K., Yew Y.P. (2020). Recent developments in the facile biosynthesis of gold nanoparticles (AuNPs) and their biomedical applications.. Int. J. Nanomedicine.

[5] Noah N. (2019). Green synthesis, characterization and applications of nanoparticles..

[6] Menon S. (2017). S R, S VK. A review on biogenic synthesis of gold nanoparticles, characterization, and its applications.. Resource-Efficient Technol.

[7] Boomi P., Ganesan R., Prabu Poorani G. (2020). Phyto-Engineered gold nanoparticles (AuNPs) with potential antibacterial, antioxidant, and wound healing activities under in vitro and in vivo conditions.. Int. J. Nanomedicine.

[8] Hu X., Zhang Y., Ding T., Liu J., Zhao H. (2020). Multifunctional Gold Nanoparticles: A novel nanomaterial for various medical applications and biological activities.. Front. Bioeng. Biotechnol..

[9] Nadeem M., Abbasi B.H., Younas M., Ahmad W., Khan T. (2017). A review of the green syntheses and anti-microbial applications of gold nanoparticles.. Green Chem. Lett. Rev..

[10] Singh P., Garg A., Pandit S., Mokkapati V., Mijakovic I. (2018). Antimicrobial effects of biogenic nanoparticles.. Nanomaterials (Basel).

[11] Folorunso A., Akintelu S., Oyebamiji A.K. (2019). Biosynthesis, characterization and antimicrobial activity of gold nanoparticles from leaf extracts of Annona muricata.. J. Nanostructure Chem..

[12] Rao P.V., Nallappan D., Madhavi K., Rahman S., Wei L.J., Gan S.H. (2016). Phytochemicals and biogenic metallic nanoparticles as anticancer agents.. Oxid. Med. Cell. Longev..

[13] Sun B., Hu N., Han L., Pi Y., Gao Y., Chen K. (2019). Anticancer activity of green synthesised gold nanoparticles from Marsdenia tenacissima inhibits A549 cell proliferation through the apoptotic pathway.. Artif. Cells Nanomed. Biotechnol..

[14] Patil MP, Kim GD (2020). Gold Nanoparticles: Biogenic synthesis and anticancer application.. Green Synthesis of Nanoparticles: Applications Prospects..

[15] Gour A., Jain N.K. (2019). Advances in green synthesis of nanoparticles.. Artif. Cells Nanomed. Biotechnol..

[16] Sharma D., Kanchi S., Bisetty K. (2019). Biogenic synthesis of nanoparticles: A review.. Arab. J. Chem..

[17] Datkhile K.D., Durgavale P.P., Patil M.N. (2017). Biogenic silver nanoparticles from Nothapodytesfoetida kill human cancer cells in vitro through inhibition of cell proliferation and induction of apoptosis.. J Bionanosci.

[18] Datkhile K.D., Patil S.R., Durgavale P.P., Patil M.N., Jagdale N.J., Deshmukh V.N. (2020). Studies on antioxidant and antimicrobial potential of biogenic silver nanoparticles synthesized using Nothapodytes foetida leaf extract (Wight) sleumer.. Biomed. Pharmacol. J..

[19] Datkhile K.D., Durgavale P.P., Patil M.N., Jagdale N.J., Deshmukh V.N. (2021). Biosynthesis characterization and evaluation of biological properties of biogenic gold nanoparticles synthesized using Nothapodytesfoetida leaf extract.. Nanosci. Nanotechnol. Asia.

[20] Datkhile K.D., Durgawale P.P., Patil M.N., Joshi S.A., Korabu K.S. (2019). Studies on phytoconstituents, in vitro antioxidant, antibacterial, antiparasitic, antimicrobial, and anticancer potential of medicinal plant Lasiosiphon eriocephalus decne (Family: Thymelaeaceae).. J. Nat. Sci. Biol. Med..

[21] Datkhile K.D., Patil S.R., Durgawale P.P. (2021). Biogenic synthesis of gold nanoparticles using Argemone mexicana L. and their cytotoxic and genotoxic effects on human colon cancer cell line (HCT-15).. J. Genet. Eng. Biotechnol..

[22] Hunyadi A. (2019). The mechanism(s) of action of antioxidants: From scavenging reactive oxygen/nitrogen species to redox signaling and the generation of bioactive secondary metabolites.. Med. Res. Rev..

[23] Nakkala J.R., Mata R., Bhagat E., Sadras S.R. (2015). Green synthesis of silver and gold nanoparticles from Gymnema sylvestre leaf extract: study of antioxidant and anticancer activities.. J. Nanopart. Res..

[24] Balashanmugam P. (2018). MosaChristas K, Kowsalya E. In vitro cytotoxicity and antioxidant evaluation of biogenic synthesized gold nanoparticles from Marselia quadrifolia on lung and ovarian cancer cells.. Int J Appl Pharmac.

[25] Chen J., Li Y., Fang G. (2021). Green synthesis, characterization, cytotoxicity, antioxidant, and anti-human ovarian cancer activities of Curcumae kwangsiensis leaf aqueous extract green-synthesized gold nanoparticles.. Arab. J. Chem..

[26] Hosny M., Fawzy M. (2021). Instantaneous phytosynthesis of gold nanoparticles via Persicaria salicifolia leaf extract, and their medical applications.. Adv. Powder Technol..

[27] Bernardos A., Piacenza E., Sancenón F. (2019). Mesoporous silica based materials with bactericidal properties.. Small.

[28] Lin A., Liu Y., Zhu X. (2019). Bacteria-Responsive biomimetic selenium nanosystem for multidrug-resistant bacterial infection detection and inhibition.. ACS Nano.

[29] Zhang J, Hurren C, Lu Z, Wang D. (2022). pH-sensitive alginate hydrogel for synergistic anti-infection.. Int J Biol Macromol.

[30] Moore J.H., Honrado C., Stagnaro V., Kolling G., Warren C.A., Swami N.S. (2020). Rapid in vitro assessment of Clostridioides difficile inhibition by probiotics using dielectrophoresis to quantify cell structure alterations.. ACS Infect. Dis..

[31] Harimoto T., Hahn J., Chen Y.Y. (2022). A programmable encapsulation system improves delivery of therapeutic bacteria in mice.. Nat. Biotechnol..

[32] Lu Z., Zhang H., Hu X., Lu J., Wang D. (2022). Probiotic-Free microfiber membrane for promoting infected wound healing by regulating wound flora balance.. ACS Materials Letters.

[33] Veziant J., Bonnet M., Occean B.V., Dziri C., Pereira B., Slim K. (2022). Probiotics/Synbiotics to reduce infectious complications after colorectal surgery: A systematic review and Meta-Analysis of randomised controlled trials.. Nutrients.

[34] Annamalai A., Christina V.L.P., Sudha D., Kalpana M., Lakshmi P.T.V. (2013). Green synthesis, characterization and antimicrobial activity of Au NPs using Euphorbia hirta L. leaf extract.. Colloids Surf. B Biointerfaces.

[35] Rahaman Mollick M.M., Bhowmick B., Mondal D. (2014). Anticancer (in vitro) and antimicrobial effect of gold nanoparticles synthesized using Abelmoschus esculentus (L.) pulp extract via a green route.. RSC Advances.

[36] Donga S., Bhadu G.R., Chanda S. (2020). Antimicrobial, antioxidant and anticancer activities of gold nanoparticles green synthesized using Mangifera indica seed aqueous extract.. Artif. Cells Nanomed. Biotechnol..

[37] Dube P., Meyer S., Madiehe A., Meyer M. (2020). Antibacterial activity of biogenic silver and gold nanoparticles synthesized from Salvia africana-lutea and Sutherlandia frutescens.. Nanotechnology.

[38] Sathiyaraj S., Suriyakala G., Dhanesh Gandhi A. (2021). Biosynthesis, characterization, and antibacterial activity of gold nanoparticles.. J. Infect. Public Health.

[39] Ismail E., Saqer A., Assirey E., Naqvi A., Okasha R. (2018). Successful green synthesis of gold nanoparticles using a Corchorus olitorius extract and their antiproliferative effect in cancer Cells.. Int. J. Mol. Sci..

[40] Wang L., Xu J., Yan Y., Liu H., Karunakaran T., Li F. (2019). Green synthesis of gold nanoparticles from Scutellaria barbata and its anticancer activity in pancreatic cancer cell (PANC‐1).. Artif. Cells Nanomed. Biotechnol..

[41] Lopez-Chaves C., Soto-Alvaredo J., Montes-Bayon M., Bettmer J., Llopis J., Sanchez-Gonzalez C. (2018). Gold nanoparticles: Distribution, bioaccumulation and toxicity. In vitro and in vivo studies.. Nanomedicine.

[42] Kus-Liśkiewicz M., Fickers P., Ben Tahar I. (2021). Biocompatibility and cytotoxicity of gold nanoparticles: Recent advances in methodologies and regulations.. Int. J. Mol. Sci..

[43] Arvindganth R., Kathiravan G. (2019). Biogenic synthesis of gold nanoparticle from Enicostema axillare and their in vitro cytotoxicity study against MCF-7 cell line.. Bionanoscience.

[44] Majoumouo M.S., Sharma J.R., Sibuyi N.R.S., Tincho M.B., Boyom F.F., Meyer M. (2020). Synthesis of biogenic gold nanoparticles from Terminalia mantaly extracts and the evaluation of their in vitro cytotoxic effects in cancer cells.. Molecules.

